# Relation of changes in productivity and remuneration of the labor factor in agriculture in EU Member States

**DOI:** 10.1371/journal.pone.0328984

**Published:** 2025-08-04

**Authors:** Zbigniew Jerzy Floriańczyk, Włodzimierz Rembisz, Aleksandra Pawłowska

**Affiliations:** 1 Institute of Agricultural and Food Economics, National Research Institute, Warsaw, Poland; 2 Department of The European Integration, Institute of Rural and Agricultural Development of The Polish Academy of Sciences, Warsaw, Poland; University of Modena and Reggio Emilia: Universita degli Studi di Modena e Reggio Emilia, ITALY

## Abstract

The article investigates whether labor productivity serves as a source for financing remuneration and whether productivity growth is adequate to support the corresponding increase in compensation within the agricultural sector. The analysis is grounded in the producer’s equilibrium, Clark’s marginal productivity theory, and the Lewis model. We utilized constant price data from the Economic Accounts for Agriculture across all EU Member States for the 2010–2022 period, encompassing the entire agricultural sector. The research methodology consisted of two stages: first, a comparative analysis of the examined categories was conducted; second, the conclusions were verified using the results of a panel regression analysis. The study reveals that, in many concerned countries, the rise in labor productivity primarily resulted from decreased agricultural employment. In specific countries, including the Czech Republic, France, Croatia, Ireland, Italy, Luxembourg, the Netherlands and Slovenia, the growth in remuneration outpaced productivity growth, suggesting potential management inefficiencies. The results of the panel regression models confirmed that labor productivity growth is strongly associated with a reduction in labor input and an increase in labor remuneration.

## 1. Introduction

### 1.1. Labor allocation theory and the productivity-remuneration relationship

In studies on competitiveness, the relationship between factor productivity and the resulting remuneration is of significant importance. These indicators are essential for effectively allocating resources among economic agents and sectors in the long term. The factors of production consist of capital (including machinery, equipment, materials, and technology), labor (considered in terms of both quantity and quality), and land (which is primarily encompassed within the capital factor). The challenge of allocating the product (or added value) among the factors contributing to its creation is a fundamental economic issue. In this context, we follow John Bates Clark’s assumption that each factor of production is compensated based on the value it contributes to the overall income of the product, specifically according to its marginal productivity [1].

In microeconomic terms, this relationship pertains to the producer’s equilibrium and is the foundation for optimizing the producer’s objective function. A producer aiming to maximize profit cannot sustain a scenario where the compensation for a factor exceeds its productivity. Such an imbalance would necessitate external financing to cover the deficit or risk bankruptcy. From a macroeconomic and sectoral perspective, this relationship is crucial for understanding allocation and distribution. If, in a particular analysis, the remuneration for a given factor of production (or multiple factors) is higher than its productivity – especially average productivity – in one or more sectors, while the reverse holds in others, it signals inefficient allocation. Conversely, this could indicate a tendency toward equalizing wage differences due to value-added transfers between sectors. This situation is inherently linked to sectoral economic efficiency and the structural changes that may accompany it, ultimately affecting the competitiveness of production within sectors. The agricultural sector occupies a unique position in this context. Historical frameworks such as the Kuznets and Lewis models [2] have highlighted that less favorable dynamics often characterize agriculture. These dynamics are indirectly reflected in the agrarian question and the foundations of agricultural policy. Thus, the well-known propositions derived from these models emphasize the necessity for structural change. In macroeconomic terms, when applied to the overall economy, adhering to the rational principle that the productivity of factors should finance their remuneration is essential for the sustainable development and competitiveness of an economy.

### 1.2. EU agriculture: institutional factors and labor productivity

The research problem addressed in the article pertains to whether the level of labor productivity serves as a source of financing its remuneration and if the productivity growth is sufficient to finance the corresponding increase in the factor’s remuneration. Initially, the issue of distinguishing factor remuneration in agriculture was associated with the dual nature of an economy with an unlimited labor supply [3]. This assumed that labor remuneration was determined by providing a living wage rather than by labor productivity, considering social norms, and institutional or non-market factors [4]. Over time, this approach has been found to be unfounded, mainly due to the limited labor movement between sectors [2,5,6]. This limited movement may play a significant role in explaining the variation in labor productivity and its remuneration across countries [7–10].

The research approach in the article is based on the producer’s choice mechanism, which aims to maximize the producer’s objective function. The producer achieves maximum profit when the remuneration of a factor of production is no greater than its marginal productivity, which equals its average productivity, at the given product price. In the case of the agricultural producer (farm) this assumption is met because they have no influence on the price level of their product in a competitive equilibrium. Simultaneously the labor remuneration is to be financed by its average productivity at a given product price. Empirical studies show variations in the relationship between remuneration and labor productivity depending on the sector or country. Productivity, especially labor productivity, is responsible for income variations, according to [11–13]. Bacsi, David and Hollosy [14], Kołodziejczak [15] and Dokić et al. [16] demonstrate a higher variability of labor productivity in agriculture compared to other sectors. This arises from the distinctive nature of agriculture and may persist even in its industrialized form, especially in the European model [17–19]. Lagakos and Waugh [20] attribute this productivity gap to the self-selection of heterogeneous workers, which determines the productivity of this sector. Cai and Pandey [21] attribute the labor productivity gap between agriculture and other sectors to likely mismeasurement of value added and sectoral differences in human capital rather than the misallocation of factors of production [22]. Herrendorf and Schoellman [23] point to technological backwardness, lack of investment, low levels of education, deagrarianization, limited market access, an inefficient institutional environment, natural conditions, and migration and demographic change. Gollin, Parente and Rogerson [24] and Restuccia, Yang and Zhu [25] find that developed countries achieve higher labor productivity than developing countries due to better access to technology, higher investment, and more efficient institutions. They also note that bridging labor productivity differences between countries is less effective in agriculture due to technology transfer costs, trade barriers, and political barriers [26,27]. Labor productivity in agriculture varies significantly among EU Member States, with Eastern European countries typically exhibiting the lowest levels [28,29]. While convergence occurred in countries with low and medium productivity, divergence was noted among those with high productivity [30].Hayami and Ruttan [31] highlighted that remuneration disparities between the agricultural and non-agricultural sectors should lead to labor movement from agriculture to other sectors, resulting in a convergence of remuneration and productivity across sectors [32–34]. However, this does not always occur, leading to an inefficient allocation of productive resources. Vollrath [35] suggested that approximately one-third of the income gap between agricultural and non-agricultural sectors can be attributed to the inefficient allocation of resources. Caselli and Coleman [36], Castellano-Álvarez et al. [37] and Ahlmeyer and Volgmann [38] identified the lack of alternative employment opportunities in rural areas as a cause of overemployment in agriculture. Additionally, Gutierrez [27] and Lagakos and Waught [20] emphasized the significance of food security and the impact of agricultural subsidies and intervention.

### 1.3. Sectoral convergence and divergence: research focus and analytical approach

Recent analyses of labor productivity, especially concerning the remuneration of this factor, are limited. Our article addresses this research gap by analyzing these categories in real terms, focusing on the entire agricultural sector across all EU Member States. We adopt the concept of Unit Labor Costs (ULC) analysis, which refers to the average labor costs per unit value of output as defined by the OECD [39]. Enhancements in ULCs are considered a way of strengthening the international competitiveness of economies. However, the correlation between changes in ULCs and shifts in competitiveness may temporarily be influenced by exchange rate policy, which is assumed to be of secondary importance in this research [40]. The article’s focus is on researching the ratio, both statically and dynamically, between labor productivity and its remuneration. First, a comparative analysis of the examined categories was conducted; second, the conclusions were validated using the results of a panel regression analysis. The article poses the question of whether the level of labor productivity is a source of financing its remuneration and whether the growth of this productivity is sufficient to finance the corresponding remuneration growth. The research objective does not hypothesize the magnitude of the ratio of the level and dynamics between productivity and labor remuneration. It only assumes that a given relation (smaller or greater than unity) between these categories may indicate more or less rational management processes, including competitiveness, in the agriculture of the European Union (EU) countries. The consideration is conducted on the grounds of positive economics. However, the problem outlined does not relate to the issue of income parity, which is a normative problem. Thus, labor productivity is not related to income parity, customarily defined by the level of income obtained in other sectors of the national economy.

## 2. Materials and methods

### 2.1. Theoretical assumptions

The first step in tackling the research problem is formally analyzing the change in labor productivity. The initial definition of labor productivity, in the context of its role as a factor influencing the volume of production, is as follows:


$$Y=\frac{Y}{L}*L$$ (1)

where: Y – output (volume) expressed in constant prices, L – labor inputs. Differentiating with respect to time gives the following result:


$$\frac{d}{dt}Y=\frac{d}{dt}Y-\frac{d}{dt}L+\frac{d}{dt}L$$ (2)

which we write as:


$$y_{t}=p_{t}+l_{t}$$ (3)

and we transform into a form that enables us to analyze changes in labor productivity over time:


$$p_{t}=y_{t}-l_{t}$$ (4)

where: $p_{t}$ – labor productivity growth over time, $y_{t}$ – production growth over time, $l_{t}$ – labor growth over time. The rise in production volume and labor input determines the increase in labor productivity in formula (4).

The research problem addressed in the article focuses on the relation between changes in labor productivity and its remuneration. This can be ascertained using producer choice theory from microeconomics. According to this theory, equilibrium is reached when the productivity of a specific factor (in this case, labor) at given product prices covers the remuneration of this factor [41]. This means equality of productivity at constant prices ($P_{Y}$) and the remuneration of the labor ($W_{L}$):


$$W_{L}\;=\frac{Y(P_{Y})}{L}$$ (5)

Differentiating with respect to time yields the following result:


$$\frac{d}{dt}W_{L}=\frac{d}{dt}Y-\frac{d}{dt}L$$ (6)

which we write as:


$$w_{t}=y_{t}-l_{t}$$ (7)

The idea that labor productivity and remuneration are equal is hypothetical and comes from the producer equilibrium mentioned earlier. There are different possible relationships here that can be interpreted in terms of the rationality of economic processes:

$w_{t}=y_{t}-l_{t}$ or $w_{t}>y_{t}-l_{t}$ or $w_{t}<y_{t}-l_{t}$ (8)

When remuneration surpasses productivity, it can trigger an irrational process, potentially diminishing the competitiveness of the producer or sector involved. This can increase agricultural prices, benefiting producers but reducing consumers’ income. Conversely, if remuneration falls below productivity, it may indicate negative distributional processes, suggesting that the price level of agricultural products is too low and draining the income of agricultural producers.

### 2.2. Data and methods

The empirical analysis used data from European agricultural production performance statistics, specifically the Economic Accounts for Agriculture (EAA) and farm indicators (farm type, economic size in Standard Output, Utilized Agricultural Area and agricultural labor input) from Eurostat databases. These variables were analyzed over the 2010–2022 period, using 2010 constant prices. The EAAs cover the entire agricultural sector and are compiled using a standardized methodology developed by Eurostat, allowing for comparisons of the economic situation of agriculture across EU countries. However, while the EAA data offer comprehensive coverage of the entire agricultural sector, their aggregated nature limits the ability to analyze specific causal relationships or detailed determinants of productivity and remuneration. This means that micro-level nuances and complexities may not be fully captured, potentially oversimplifying some of the intricate dynamics within the sector.

The research conducted a comparative analysis of agricultural production, labor input, labor productivity and labor remuneration to investigate the relationship among these variables in 27 EU Member States, referencing the producer’s equilibrium, Clark’s theory of marginal productivity, and the Lewis model.

### 2.3. Model specification

In the final step of the analysis, a panel regression model was estimated based on balanced panel data with 324 observations, encompassing 27 EU Member States over the period 2011–2022, to determine the relationship between labor remuneration (income), labor productivity and labor input. The models estimated are as follows:

Model 1:


$${\left(\Delta \frac{Y}{L}\right)}_{it}=\alpha +\beta {\Delta L}_{it}+\varepsilon_{it}$$ (9)

where: $\Delta \frac{Y}{L}$ – change in labor productivity, ΔL – change in labor input.

Model 2:


$${\left(\Delta W_{L}\right)}_{it}=\alpha +\beta \Delta L_{it}+\varepsilon_{it}$$ (10)

where: $\Delta W_{L}$ – change in labor remuneration (income), ΔL – change in labor input.

Model 3:


$${\left(\Delta W_{L}\right)}_{it}=\alpha +\beta {\left(\Delta \frac{Y}{L}\right)}_{it}+\varepsilon_{it}$$ (11)

where: $\Delta W_{L}$ – change in labor remuneration (income),$\;\Delta \frac{Y}{L}$ – change in labor productivity.

The pooled Ordinary Least Squares regression was used based on the results of the Chow test [42], the Hausman test [43] and the Lagrange Multiplier test [44].

### 2.4. Software

The study was conducted using R [45] and package plm [46].

## 3. Results

### 3.1. Farm structure across EU member states

**Fig 1:**
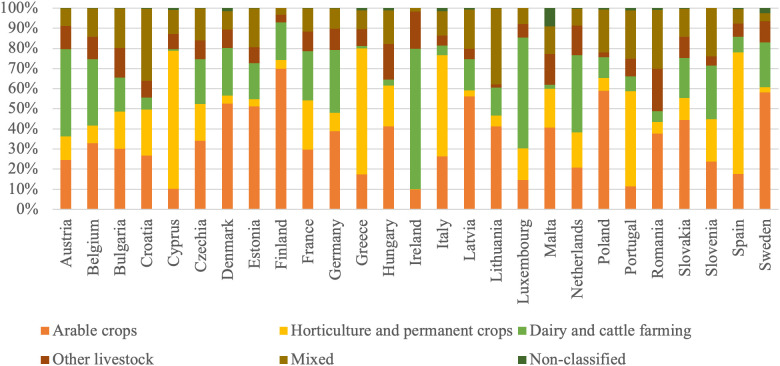
Distribution of farm types by EU Member States in 2020 (proportion of the total farms).

**Fig 2:**
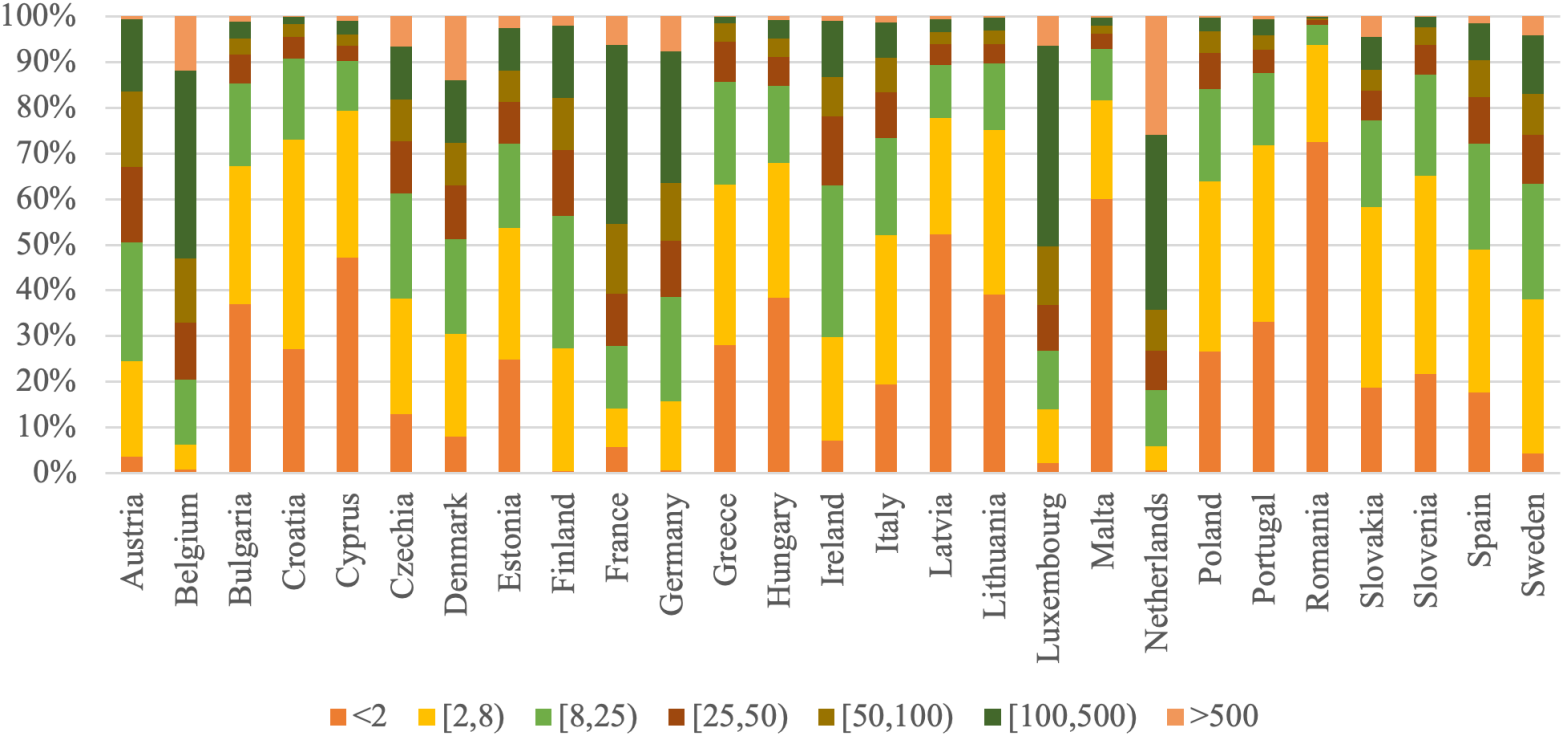
Distribution of economic size classes by EU Member States in 2020 (proportion of the total farms).

**Fig 3:**
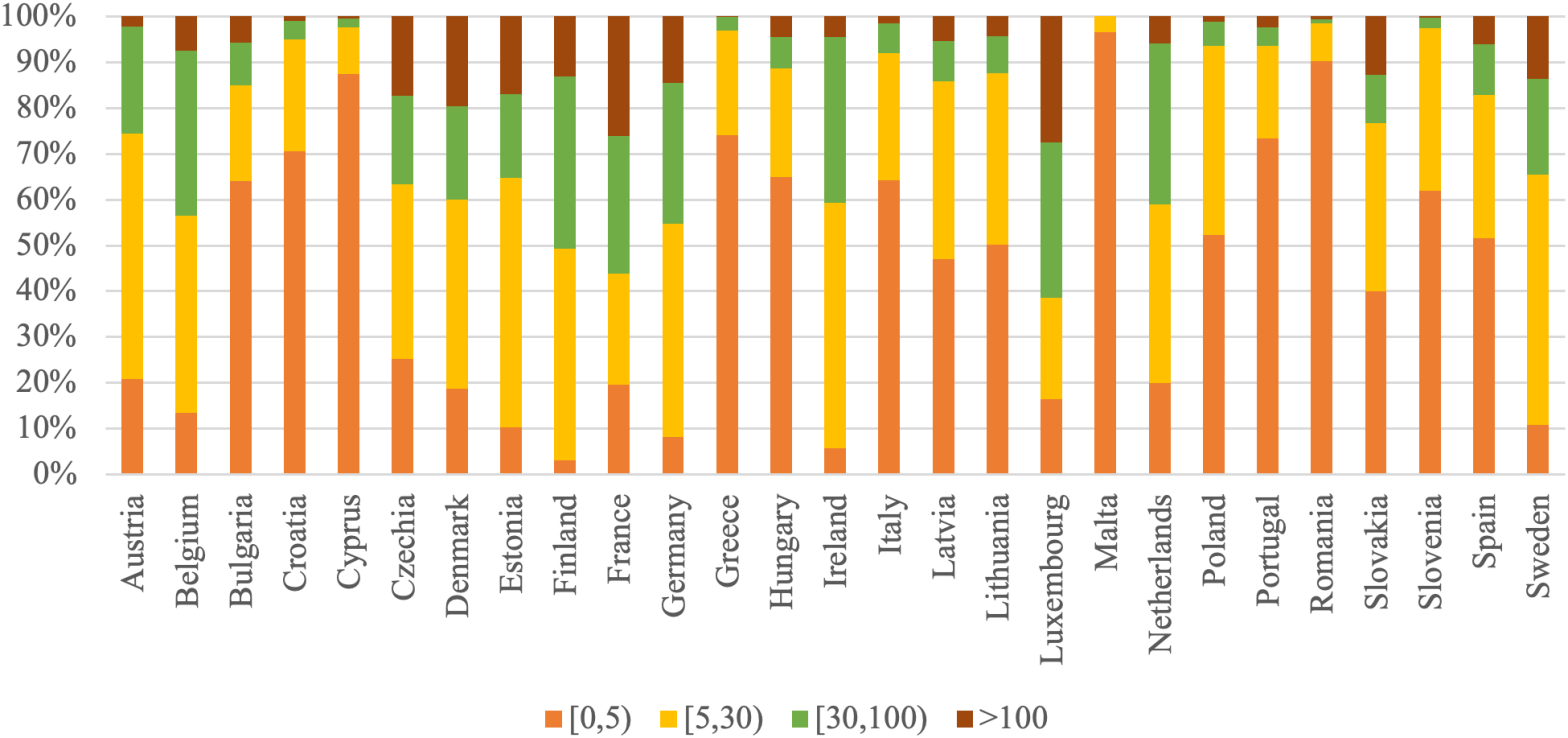
Distribution of Utilized Agricultural Area by EU Member States in 2020 (proportion of the total farms).

To provide a clearer perspective on the relationships between changes in productivity and remuneration of the labor factor in agriculture, the analysis includes a comparison of agrarian structures across EU countries. Accordingly, Figs 1–3 present the distribution of farm specialization types, economic sizes, and utilized agricultural area among farms, highlighting the diversity of structural patterns within the EU Member States.

Finland has the highest proportion of arable crop farms at 69.9%, far above the European average, while Cyprus (10.2%) and Ireland (9.8%) have the lowest shares. In horticulture and permanent crops, Cyprus (68.7%) and Greece (62.7%) are prominent, whereas Ireland (0.2%) has an almost nonexistent contribution. Dairy and cattle farming are prevalent in Ireland (69.8%) and Luxembourg (55.9%), but Cyprus (0.8%) and Greece (1.2%) participate minimally. For mixed farming, Lithuania (37.9%) and Croatia (35.8%) are leading, while Ireland (1.6%) and Sweden (3.8%) show the smallest shares. Countries like Czechia, Belgium, and Germany tend to have more balanced agricultural profiles, with significant shares across various farm types, indicating diverse farming systems.

Romania has the highest percentage of very small farms (less than EUR 2,000 Standard Output), making up 72.5% of its total, with Malta (60.0%) and Latvia (52.3%) close behind. In contrast, the Netherlands (0.7%), Germany (0.6%), and Finland (0.5%) have very low proportions in this smallest category. The economic size class of EUR 2,000–8,000 Standard Output is predominantly observed in Croatia (45.9%), Slovenia (43.3%), and Portugal (38.7%), whereas its prevalence is significantly lower in Belgium (5.4%) and the Netherlands (5.3%). Farms with an economic size between EUR 8,000 and 25,000 Standard Output are notably prevalent in Ireland (33.3%), Austria (25.9%), and Sweden (25.4%), while Romania has the smallest share at 4.5%. Mid-sized farms (EUR 25,000–100,000 Standard Output) have notable shares in Austria, Ireland, and Finland, each exceeding 25%. For the largest farm categories (more than EUR 500,000 Standard Output), the Netherlands has the largest share at 26.0%, followed by Belgium (11.9%) and Denmark (14.0%). Greece (0.1%) and Romania (0.1%) have almost negligible shares in this size class.

In the smallest farm size category (0–5 ha), Malta (96.6%), Romania (90.3%), Cyprus (87.5%), and Greece (74.1%) show exceptionally high proportions, highlighting a dominance of very small agricultural holdings. In contrast, Finland (3.1%), Ireland (5.7%), and Germany (8.2%) have significantly lower percentages in this group. In Sweden (54.7%), Estonia (54.3%), Ireland (53.6%), and Austria (53.7%), intermediate-sized farms (5–30 ha) make up the majority. Conversely, Malta (3.4%), Cyprus (10.1%), and Romania (8.2%) have the smallest share in this category. Larger farms (30–100 ha) hold significant shares, especially in Finland (37.7%), Ireland (36.2%), the Netherlands (35.1%), Belgium (35.9%), and Luxembourg (34.0%). This size category is rarely represented in Malta (0.0%), Romania (1.0%), Cyprus (1.9%), and Greece (2.8%). Finally, the largest farms (over 100 ha) are especially common in Luxembourg (27.7%), France (26.1%), and Denmark (19.7%), while they are very rare in Malta (0.0%), Greece (0.2%), Slovenia (0.2%), and Romania (0.6%).

The agrarian structure across EU countries is highly diverse, with Western and Northern European countries (such as Finland, Ireland, the Netherlands, and Luxembourg) notably characterized by larger and more economically robust farms, often specializing in arable crops or dairy production. Finland stands out with the highest share of arable crop farms (69.9%), while Ireland leads in dairy and cattle farming (69.8%). In contrast, Southern and Eastern European countries like Romania, Malta, Cyprus, and Greece are dominated by small-scale holdings, both in terms of land area and economic size, with very high shares of farms below 5 ha (up to 96.6% in Malta and 90.3% in Romania) and very small economic output (such as 72.5% of Romanian farms generating less than EUR 2,000 Standard Output). Countries like Cyprus and Greece also have the highest shares of farms specializing in horticulture and permanent crops. Meanwhile, countries like Belgium, Germany, and Czechia have a more balanced agricultural structure, with diverse farm types, economic classes, and land sizes.

### 3.2. Comparative analysis

**Fig 4:**
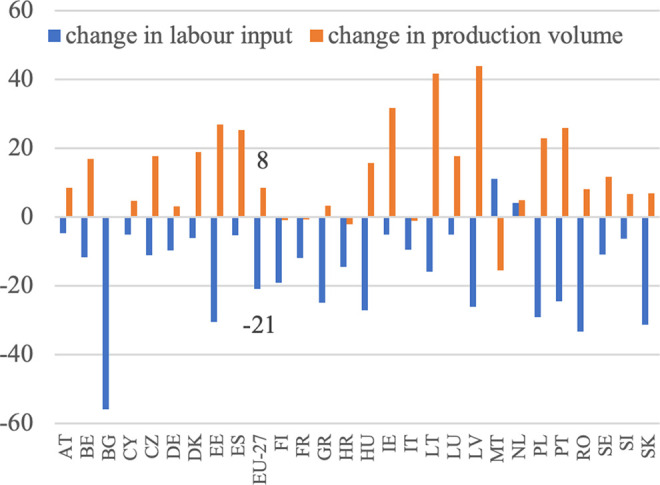
Production and labor input dynamics in agriculture (%).

**Fig 5:**
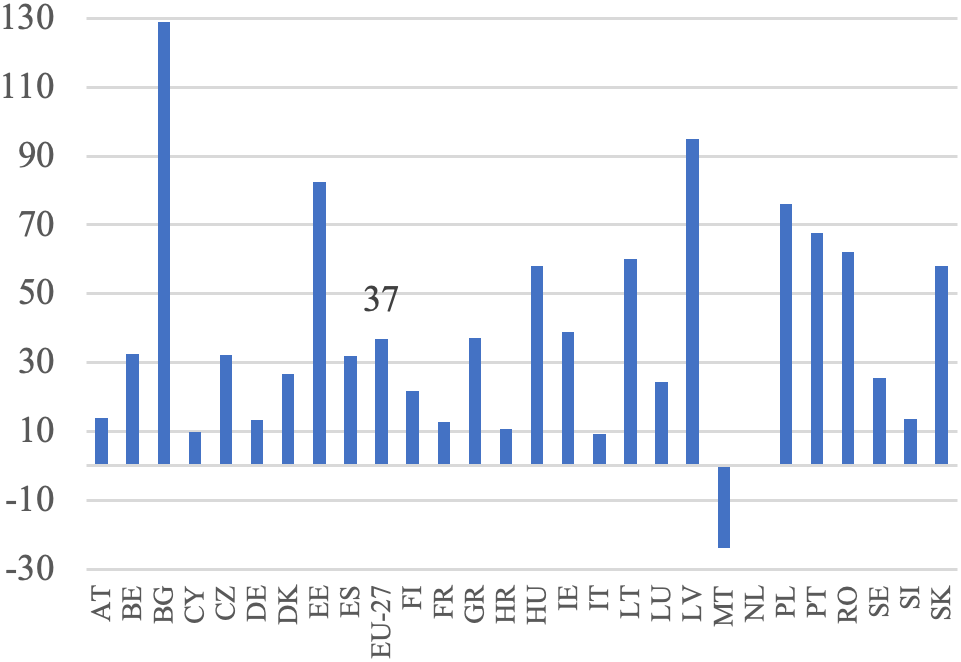
Labor productivity dynamics (%).

**Fig 6:**
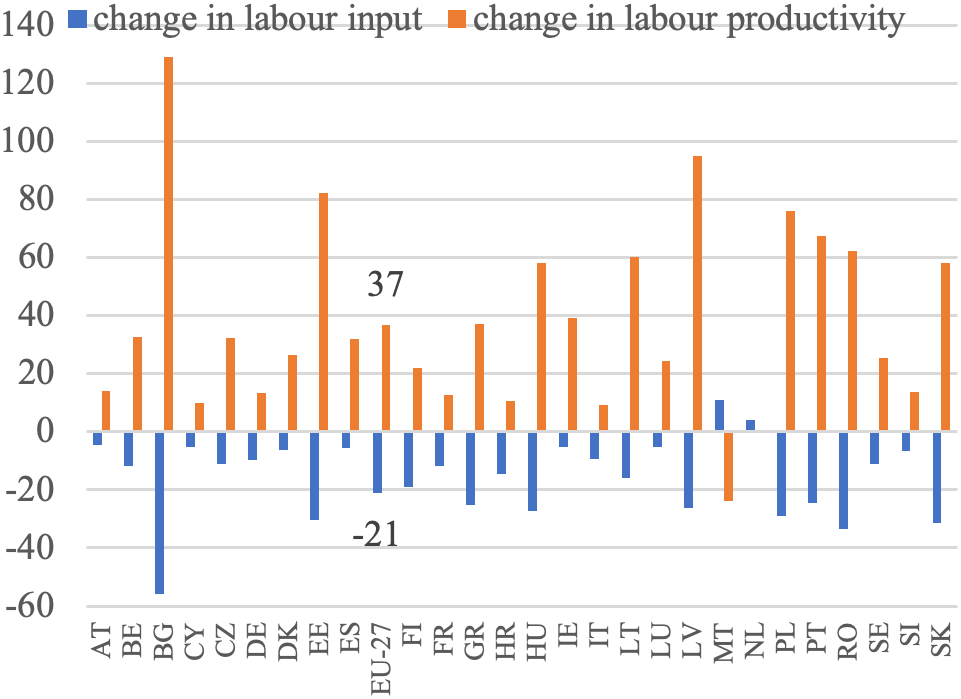
Labor productivity and labor input dynamics (%).

**Fig 7:**
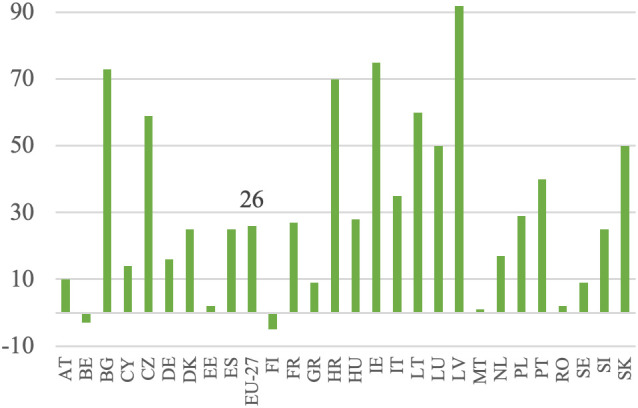
Labor remuneration dynamics (%).

**Fig 8:**
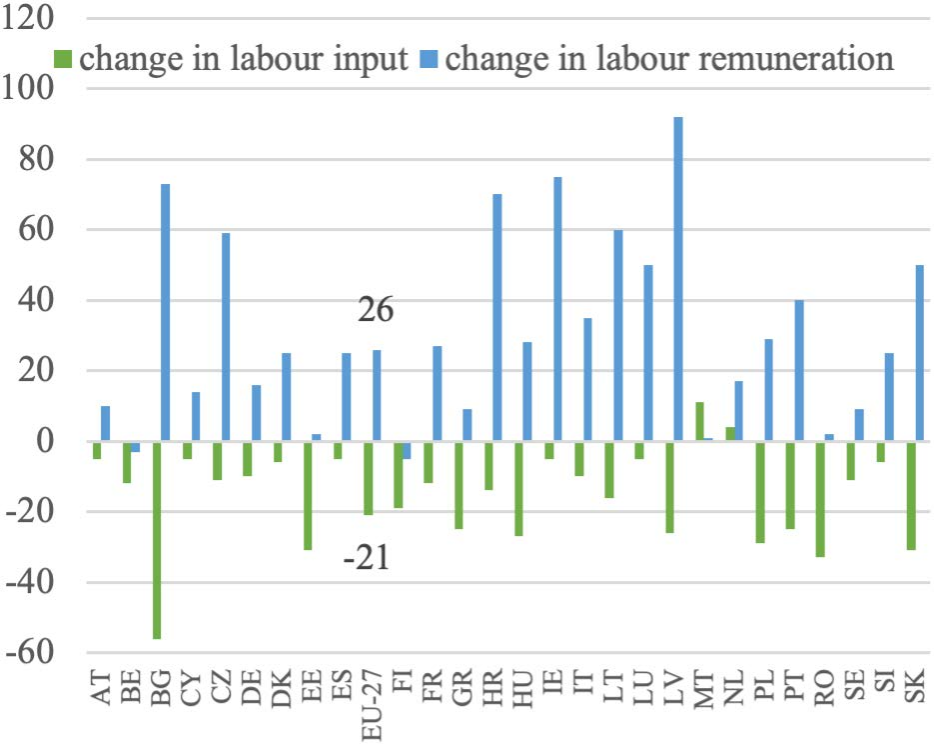
Labor remuneration and labor input dynamics (%).

**Fig 9:**
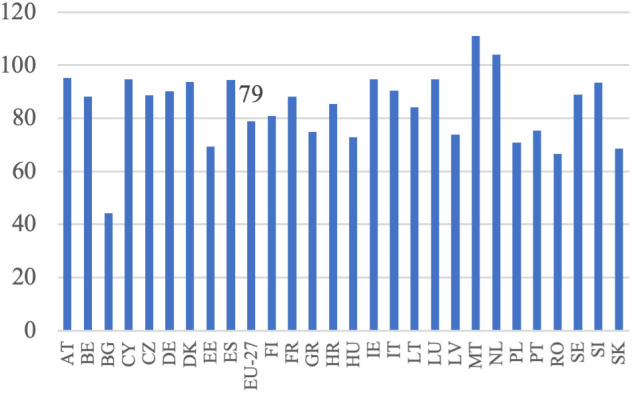
Contribution of labor input change to labor productivity growth (%).

**Fig 10:**
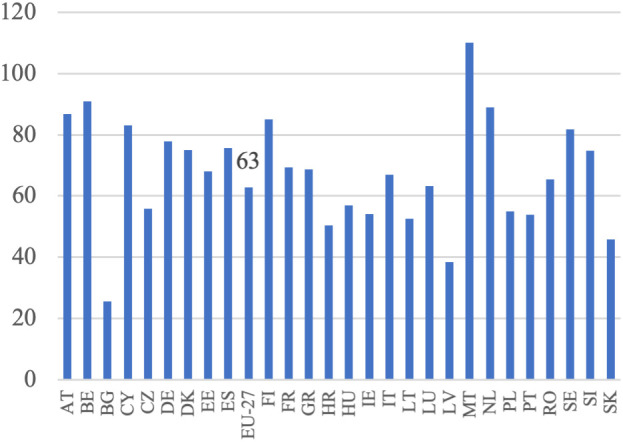
Contribution of labor input change to remuneration growth (%).

**Fig 11:**
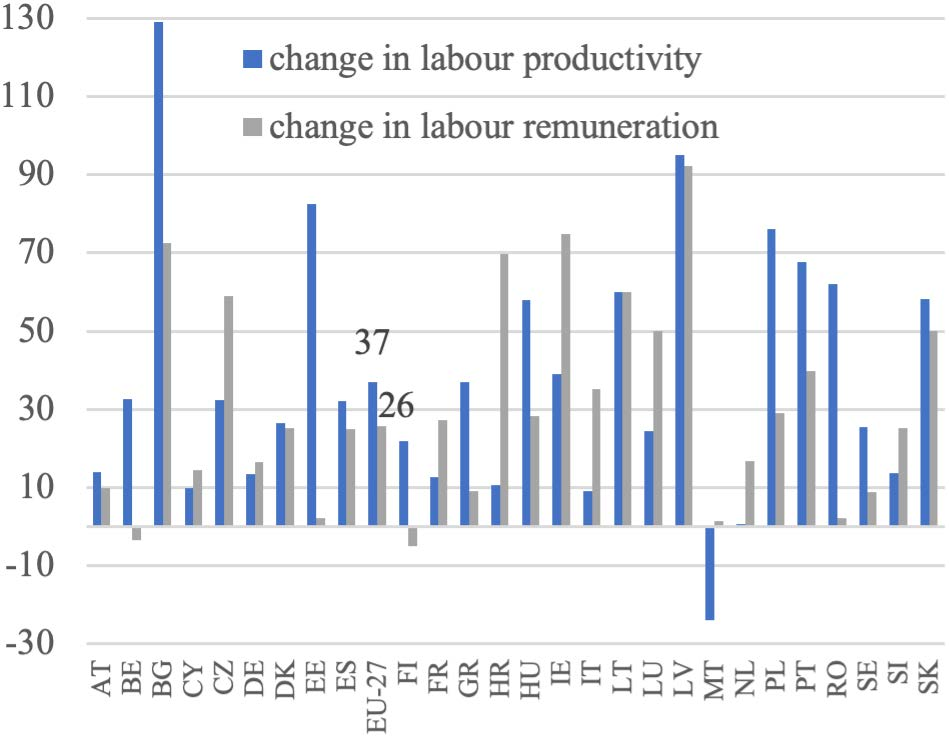
Labor productivity and labor remuneration dynamics (%).

**Fig 12:**
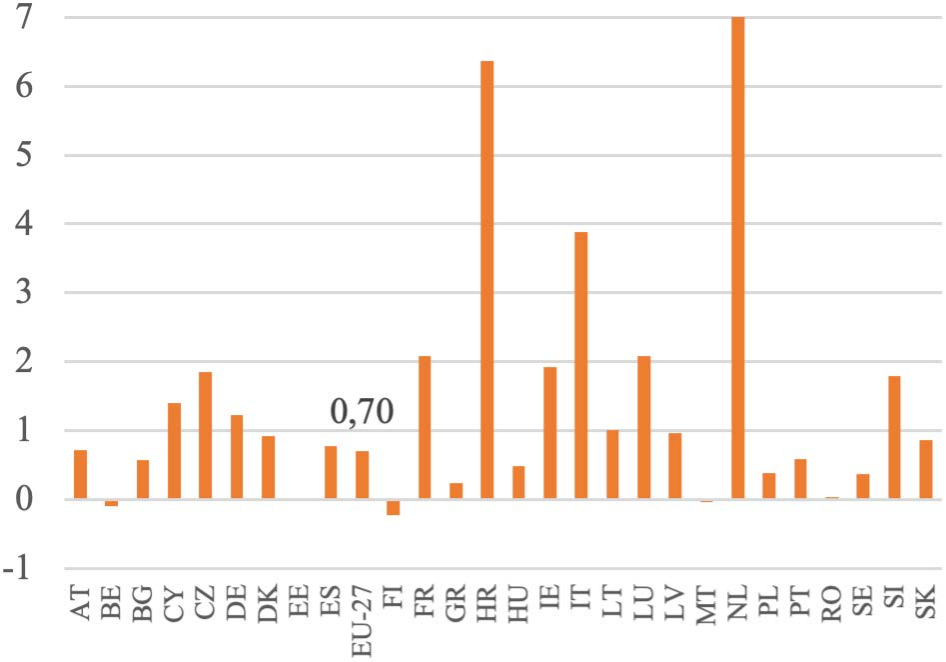
Relation of labor remuneration dynamics to labor productivity dynamics (%) Notes: Change calculated as the ratio of the average value of the variable for the period 2020–2022 to the average value of this variable for the period 2010–2012, in 2010 constant prices. Source: Eurostat, Economic Accounts for Agriculture.

The first step in the analysis, as outlined in formula (4), is to empirically illustrate the changes in production volume and labor input in the agriculture of EU countries between the two periods under consideration (see Figs 4–12). Based on this formula’s logic, displaying the dynamics of selected characteristics of agricultural sector in EU Member States on separate graphs allows us to emphasize the importance of the ratio of these changes in shaping labor productivity growth.

Based on equation (4), it was assumed that an increase in the volume of production should be accompanied by a decrease in the employment of the labor force to achieve higher labor productivity. This increase in productivity would be derived from these two sources. This trend has been observed in all EU countries, except Malta, the Netherlands, and Bulgaria, as illustrated in Fig 4. This reflects the ongoing development of European agriculture, specifically the growth model of agriculture. Additionally, the decrease in employment appears to have been the primary driver of the increase in labor productivity in EU agriculture during this period. This is particularly noticeable in countries that joined the EU later and previously had centrally planned economies. In these countries, such as Bulgaria, Romania, Poland, Slovakia, Hungary, and Estonia, agricultural employment declined significantly, indicating rapid adjustments to EU farming conditions and competition in the common market.

In Western European countries, where structural changes occurred in the 1980s, agriculture experienced a slight decline in labor input. During the analyzed period, the EU countries with the highest production growth were Lithuania, Latvia, Estonia, Poland, Ireland, Spain, and Portugal. Conversely, Malta, Croatia, Finland, Bulgaria, Sweden, Italy, and Romania experienced a decrease in production volume. Notably, among the major agricultural producers in the EU, namely France, Germany, Spain, Italy, and Poland, the trends vary significantly. France and Italy experienced slight decreases in production volume (−1%) despite reductions in labor input (−12% and −10%, respectively), which may reflect structural saturation. In contrast, Germany achieved a moderate production increase (+3%) with a 10% labor input reduction, and Spain showed strong growth (+25%) with only a 5% labor input decline. Poland stands out with a substantial increase in production volume (+23%) accompanied by a sharp decrease in labor input (−29%). According to equation (4), summing the aforementioned percentage increases in agricultural production volume and decreases in labor input resulted in indicators of productivity changes, as shown in Fig 5. Throughout the analyzed period, there was considerable variation in the growth of labor productivity in agriculture across EU countries, with Malta being the only exception where this indicator turned negative. The highest productivity growth was observed in Bulgaria (+129%), Latvia (+95%), Estonia (+82%), Poland (+76%) and Romania (+62%), all from Eastern Europe (excluding Portugal). This suggests that these countries may be compensating for previous differences in productivity levels, with higher growth resulting from a lower base, often referred to as backwardness rent. A similar dynamic was evident in other new Member States, such as Lithuania (+60%), Hungary (+58%), and Slovakia (+58%), where rapid structural adjustment and modernization led to marked improvements in productivity. By contrast, in the so-called old EU countries, where such adjustments occurred earlier and agriculture is already more capital-intensive, growth rates were typically lower. Countries like France (+13%), Germany (+13%), and Italy (+9%) exhibited modest changes, reflecting a more saturated productivity potential. Some older Member States nonetheless recorded stronger gains—Portugal (+68%) and Ireland (+39%) being notable examples—likely due to continued restructuring or efficiency gains in specific subsectors. At the other end of the spectrum, the Netherlands saw almost no productivity growth (+1%), while Malta experienced a decline (–24%) due to an increase in labor input accompanied by a fall in output. In agricultural economic theory and agricultural development models, there is a substitution-compensation relationship between the increase in labor productivity and the decrease in labor involvement. The extent of this substitutability can be visualized (without specifying the direction and strength of causality), as illustrated in Fig 6. The negative relationship between changes in labor input and changes in labor productivity indicates a substantial impact of reductions in labor input on increases in labor productivity. This implies that the increase in labor productivity nearly completely compensates for the decrease in labor input. This pattern is observed in most EU Member States, except for Malta. The data show that in the majority of countries, substantial reductions in labor input were accompanied by notable increases in labor productivity. The clearest examples of this compensatory mechanism are found in Bulgaria (−56% labor input, + 129% productivity), Latvia (−26%, + 95%), Estonia (−31%, + 82%), Poland (−29%, + 76%), and Romania (−33%, + 62%). Similarly, Hungary, Slovakia, and Lithuania, which also experienced labor declines of over 15%, achieved strong productivity growth (between +58% and +60%). In contrast, countries with smaller labor input reductions (such as Austria, Cyprus, and Luxembourg) recorded more moderate productivity increases, while the Netherlands and Malta stand out as outliers. In the Netherlands, labor input increased slightly (+4%), and productivity gains were minimal (+1%). In Malta, the rise in labor input (+11%) coincided with a decline in productivity (–24%), making it the only EU country with a clear inverse pattern. While it is generally assumed that an increase in labor productivity, resulting from technological advancements and technical progress, offsets the loss of labor input, the opposite scenario can also occur. For example, a decline in labor input due to heightened demand for labor in other sectors can drive up labor productivity in agriculture, thanks to the expansion of non-agricultural sectors in the economy. This aligns with the theories of Lewis [3] and Todaro [47].

In line with the research objective, it is crucial at the initial stage of the analysis to determine the growth of labor remuneration. This is defined as income at constant prices per full-time employee. Labor remuneration data is adjusted to exclude the influence of subsidies, the amount of which is a direct result of the policy applied. The examination of Fig 7 reveals significant variation in the growth of labor remuneration across EU countries’ agricultural sectors. This trend is clearly visible in the data, where Central and Eastern European countries such as Latvia (+92%), Bulgaria (+73%), Croatia (+70%), Lithuania (+60%), Slovakia (+50%) and the Czech Republic (+59%) recorded some of the highest increases. These changes point to a process of gradual convergence in income levels. Poland (+29%) and Hungary (+28%) also surpassed the EU-27 average (+26%), further supporting the broader pattern. Among older Member States, growth was more moderate, with Ireland (+75%) and Portugal (+40%) standing out as exceptions. In contrast, countries like Belgium (−3%) and Finland (−5%) recorded negative changes, while others, including Austria (+10%), Germany (+16%), and the Netherlands (+17%), showed relatively limited remuneration dynamics. The weakest positive change was observed in Malta (+1%) and Estonia (+2%). This variation is influenced by factors such as the agrarian structure, the capital-labor ratio, and the growth patterns in each Member State’s agriculture. Notably, countries that joined the EU after 2004 generally experienced faster growth in labor remuneration compared to the EU-27 average for agriculture.

In Fig 8, the relationship between the growth of labor remuneration and the decrease in labor input is illustrated based on equation (4). This relationship is also recognized in the context of agrarian economics, where a reduction in employment is considered decisive for increasing labor remuneration. This concept is particularly emphasized in the Lewis model and the agrarian issue [48,49]. Although the Member States are currently in a different development stage than when these models were formulated, the decline in employment is still viewed as the key factor contributing to higher labor remuneration for those employed in agriculture. Upon comparing the growth in labor remuneration with the decline in labor input and referring to equation (7), it becomes evident that these figures can be combined to a certain extent in most cases, except Malta. The clearest examples include Bulgaria (−56% labor input, + 73% remuneration), Slovakia (−31%, + 50%), Latvia (−26%, + 92%) and Lithuania (−16%, + 60%), where deep employment cuts coincided with substantial remuneration growth. A similar pattern was visible in Poland (−29%, + 29%) and Portugal (−25%, + 40%), which is particularly relevant given their fragmented agrarian structures. At the EU-27 level, a decline in labor input by 21% corresponded with a 26% rise in remuneration, reinforcing the notion of substitution-driven income growth. However, this relationship was not universal. In Malta (+11%, + 1%), labor input increased with negligible remuneration growth, while in the Netherlands (+4%, + 17%) and Finland (−19%, −5%), the patterns diverged from theoretical expectations. Moreover, in some countries with modest declines in labor input, such as Austria (−5%, + 10%) or Cyprus (−5%, + 14%), remuneration still grew moderately, suggesting other sources of this growth.

In the earlier sections of the analysis, we referred to the sources of productivity and labor remuneration growth. Our inferences are based on the analysis of Figs 9 and 10. According to the principles of agricultural economics, it is assumed that the primary source of labor productivity growth may be its decline with an increase in production levels. An increase in labor productivity can occur with a rise in labor input and an even greater increase in production levels. Fig 11 illustrates that the baseline scenario is predominant, except Malta and the Netherlands in agriculture. The share of the decrease in labor input in the increase in productivity ranged from about 45 to almost 90%, indicating the regularity of this period of agricultural development. The analysis of Fig 9 leads to the general conclusion that the decrease in employment has a significant impact on the productivity growth of the labor factor. In most Member States, except Bulgaria, the decline in employment contributed to over 50% of the productivity growth in agriculture. This suggests that changes in the agrarian structure continue to play a crucial role in enhancing labor productivity in European agriculture. The reduction in labor input is a consequence of these structural changes. This trend has persisted into the twenty-first century, despite expectations that its significance would diminish. This persistence may be attributed to the effects of the Common Agricultural Policy (CAP), particularly the income support provided through hectare-based direct payments, which has maintained the agrarian structure. Additionally, Fig 9 indicates that the role of production growth in improving labor productivity is relatively minor, possibly due to constraints on the demand for agricultural products in Europe during the analyzed period.

The analysis of Fig 10 provides the foundation for making similar comments concerning the influence of decreasing employment on the growth of labor remuneration. The decline of labor input as a source of remuneration growth in agriculture in the analyzed countries varies significantly. It ranges from 26% in Bulgarian agriculture to approximately 80–90% in the agriculture of Belgium, Austria, the Netherlands, or Finland. Generally, this decline is a notable factor in remuneration growth in agriculture in EU countries. The only exception is agriculture in Malta, where increased labor input resulted in decreased remuneration.

In line with the study’s objective, the analysis compared the increases in remuneration and labor productivity, as depicted in Fig 11. If the assumption regarding the equality of the two indicators, as stated in the methodological section, were valid, then the remuneration and labor productivity increase should be equal. However, this is not the case. This suggests that labor remuneration relative to its productivity may have increased at a slower or faster rate. In certain countries, the growth in remuneration in the agricultural sector has significantly surpassed the growth in labor productivity. These countries include the Czech Republic, France, Croatia, Ireland, Italy, Luxembourg, the Netherlands, and Slovenia. Conversely, labor productivity growth has outpaced remuneration growth in other countries, including Austria, Belgium, Bulgaria, Denmark, Estonia, Spain, Finland, Greece, Hungary, Latvia, Poland, Portugal, Romania, Sweden, and Slovakia. Overall, there appears to be a correlation between these figures, with productivity growth often exceeding labor remuneration growth. This suggests a degree of rationality in management practices in this regard.

Fig 12 presents figures calculated using the concept of unit labor costs, aiming to draw broad conclusions in the study. These figures illustrate the ratio between the growth in labor remuneration and the growth in labor productivity. A ratio greater than 1 suggests that labor remuneration is increasing faster than productivity, potentially indicating management inefficiency or other sources of income growth unrelated to CAP transfers. These additional sources may include land rents, depreciation of physical capital (such as machinery and equipment), and purchase prices higher than actual production costs, in addition to productivity gains. The analyzed indicator, which ideally should be close to or below 1, is met by the agriculture sectors of several EU countries. Particularly low ratios were observed in Romania (0.03), Estonia (0.02), Belgium (−0.09), Finland (−0.23), and Malta (−0.04). These values may reflect not only efficiency, but also stagnation or statistical anomalies due to minimal productivity gains. Countries such as Austria (0.71), Spain (0.78), Slovakia (0.86), Denmark (0.93), and Latvia (0.97) remained close to the EU-27 average (0.70), indicating a relatively balanced relationship between remuneration and productivity. Central and Eastern European countries like Bulgaria (0.57), Hungary (0.48), Poland (0.38), Lithuania (1.00), and Portugal (0.59) also fall into this efficiency range. By contrast, several Member States recorded significantly higher ratios, particularly the Netherlands (17.00), Croatia (6.36), Italy (3.89), France and Luxembourg (2.08 each), Ireland (1.92), Czechia (1.84), and Slovenia (1.79). **3.3. Panel model**

Table 1 presents the results of the estimated panel regression models, which examine the interrelationships among labor remuneration (income), labor productivity, and labor input.

**Table 1:** Results of pooled OLS models, n = 324.

Model 1 – Dependent variable: Labor productivity					
Effect	Estimate	SE	95% CI		p
			LL	UL	
Intercept	0.013	0.004	0.005	0.020	0.001
Labor input	−0.988	0.088	−1.161	−0.816	<0.001
R-Squared: 0.28273					
Adj. R-Squared: 0.2805					
F-statistic: 126.923 on 1 and 322 DF, p-value: < 0.001					
**Model 2 – Dependent variable: Income**					
Effect	Estimate	SE	95% CI		p
			LL	UL	
Intercept	0.035	0.009	0.017	0.052	<0.001
Labor input	−0.620	0.200	−1.015	−0.226	0.002
R-Squared: 0.028929					
Adj. R-Squared: 0.025913					
F-statistic: 9.59257 on 1 and 322 DF, p-value: 0.0021256					
**Model 3 – Dependent variable: Income**					
Effect	Estimate	SE	95% CI		p
			LL	UL	
Intercept	0.016	0.008	0.001	0.031	0.040
Labor productivity	0.987	0.095	0.801	1.173	<0.001
R-Squared: 0.25269					
Adj. R-Squared: 0.25037					
F-statistic: 108.88 on 1 and 322 DF, p-value: < 0.001					

Source: own calculation based on the Economic Accounts for Agriculture and Eurostat data.

Model 1 revealed a significant negative relationship between change in labor input and labor productivity, β = −0.988, p < .05, indicating that higher labor input is associated with lower labor productivity. Given that the coefficient approximates unity, this observation reinforces the prior conclusion that the increase in labor productivity nearly entirely offsets the reduction in labor input. The results of model 2 are also in line with the earlier conclusions and with the broader theoretical framework of agrarian economics. The pooled OLS model revealed a significant negative relationship between labor input and labor remuneration (income), β = −0.620, p < .05, indicating that higher labor input is associated with lower labor remuneration. In particular, this negative association is well-documented in the Lewis model and the agrarian issue, which emphasize the importance of reducing employment in traditional agricultural sectors to facilitate wage growth. According to this perspective, labor migration away from low-productivity sectors can lead to increased earnings for the remaining workforce, as capital accumulation and technological advancements drive productivity gains. Finally, the panel regression analysis revealed a significant positive relationship between labor productivity and labor remuneration (income), β = 0.987, p < .05, indicating that an increase in labor productivity is strongly associated with an increase in labor remuneration. In all Member States, labor productivity increased more rapidly than labor remuneration, on average. A situation in which remuneration falls behind productivity may indicate unfavorable distribution trends, suggesting that the low prices of agricultural products are diminishing the income of agricultural producers.

## 4. Discussion

In most countries analyzed, the increase in labor productivity was primarily linked to a reduction in agricultural employment, confirming the results of previous studies conducted by Ciaian and Kancs [50], Alvarez-Cuadrado and Poschke [51], Federico and Malanima [52] and Bocean [53]. However, Malta, the Netherlands, and Bulgaria showed deviations from this trend. Throughout the study period, notable variations in labor productivity growth within the agriculture sector of EU countries were observed [54–58]. The highest productivity growth was particularly evident in Eastern European countries, which, according to Lerman [59], indicates a narrowing of previous productivity gaps. However, in the future the labour productivity growth is to be higly depended on the level of the investment of capital that is clearly observed in developed countries [60,61].

Additionally, significant discrepancies in labor remuneration were noted [62–67]. Countries where remuneration increased at a rate surpassing the EU-27 average in agriculture included those who joined the EU after 2004. In certain countries, remuneration growth outpaced labor productivity, implying potential management inefficiencies. These are mainly connected with subsdies directly supporting agricultural incomes [68–71]. This group encompassed the Czech Republic, France, Croatia, Ireland, Italy, Luxembourg, the Netherlands, and Slovenia. In countries where remuneration growth exceeded productivity gains, other income sources – such as land rents or higher purchase prices – were identified [72].

The results of the panel regression models confirmed previous findings, demonstrating that labor productivity growth is strongly associated with a reduction in labor input and an increase in labor remuneration. The negative relationship between labor input and both productivity and remuneration aligns with agrarian economic theories, reinforcing the necessity of employment reductions to enhance sectoral efficiency. Furthermore, the positive correlation between labor productivity and remuneration suggests that rising productivity generally leads to income growth, although in most Member States, productivity gains have outpaced wage increases, highlighting potential distributional concerns.

## Conclusions

Analysis of labor productivity trends revealed a consistent pattern across most EU countries, where reductions in agricultural employment notably enhanced labor productivity. This was particularly pronounced in newer Member States from Eastern Europe (Bulgaria, Latvia, Estonia, Poland and Romania), where substantial productivity improvements were attributed to significant labor input declines, reflecting rapid structural adjustments and modernization post-EU accession. In contrast, older Member States displayed more moderate productivity growth, reflecting a higher baseline of agricultural capital intensity and prior structural adjustments.

Labor remuneration exhibited substantial variation across the EU. Post-2004 Member States typically experienced higher remuneration growth rates compared to older Member States, suggesting ongoing convergence in agricultural income levels. Notable remuneration increases occurred in Latvia, Bulgaria, Croatia, Lithuania, Slovakia and the Czech Republic. Older Member States, except for Ireland and Portugal, showed comparatively modest or negative remuneration trends, highlighting different stages and dynamics of agricultural economic development.

The study revealed that in some countries – such as the Czech Republic, France, Croatia, Ireland, Italy, Luxembourg, the Netherlands, and Slovenia – remuneration growth in the agricultural sector has significantly exceeded labor productivity growth. Conversely, in Austria, Belgium, Bulgaria, Denmark, Estonia, Spain, Finland, Greece, Hungary, Latvia, Poland, Portugal, Romania, Sweden, and Slovakia, labor productivity has increased more rapidly than remuneration. Overall, labor productivity growth tends to be linked with remuneration increases, often with productivity gains surpassing income growth, which suggests rational management practices in the sector. Nevertheless, the observed discrepancies in remuneration relative to productivity emphasize the importance of considering price-setting mechanisms and structural factors influencing income distribution within the agricultural sector. Namely, agriculture and rural development policies such as CAP should reconsider further focus on subsidies as a main instrument supporting farmers’ incomes.
